# A Case of Gangrene, Following Laceration of the Popliteal Artery and Nerve

**Published:** 1853-09

**Authors:** M. Shepherd

**Affiliations:** Payson, Ills.


ART. III.—*A Case of Gangrene, following laceration of the Popliteal Artery and Nerve.* By M. SHEPHERD, M.D., of Payson, Ills.

**H. H. M.**, a Scotchman by birth, 28 years of age, and of good constitution, was thrown from his wagon, on the night of the 19th of February, 1852, and severely injured. The night being very dark, and his horses running at a rapid rate, it could not be ascertained how the injury was inflicted. His wife, and some of his neighbors, were attracted to the spot by his cries, but before attempting to remove him a messenger was started for me, a distance of four miles. I was informed that both his legs were broken. I provided myself with such things as I supposed would be called for under the circumstances, and hastened to his dwelling.

It was one o'clock, A. M., when I saw him, one hour and a half after the accident occurred. He was then lying on the floor, with his clothes saturated with blood, complaining bitterly of the cold. His friends, before I arrived, had administered whisky and camphor to him, freely, for the purpose of warming him. After removing his clothes, it was found that his legs were not broken, but badly hurt in other respects. There was a lacerated wound, from whence the blood had flown, about eight inches in length, commencing three inches below the knee, on the outside of the left leg, and extending up into the popliteal region. The origin and attachment of several muscles were broken up, the biceps flexor curis was entirely separated an inch or more above its attachment. In cleansing the wound I found the popliteal artery, also, entirely severed. The camphor and whisky which he had swallowed while lying near a hot stove, had brought about a reaction, and as soon as the artery was touched it commenced bleeding profusely. I had no instruments with me but a common pocket case; there was but a single candle in the room, and but one person, and that a lady, possessing courage enough to attempt to hold that. The artery was torn off high up, in a deep wound, and being a good deal retracted, and bleeding freely, my embarrassment under the circumstances can be better imagined than described. For the moment I was compelled to secure the artery between my thumb and finger, the jet of blood serving as a guide to its mouth. Afterwards it was clasped with a pair of straight forceps and intrusted to the lady who assisted me, until I could provide and apply a ligature, which was no easy task, situated as I was. The wound was then brought together and secured by stitches and adhesive strips.

In due time healthy granulations made their appearance, and so far as the wound itself was concerned, there was every assurance of a speedy recovery. But not so with the leg and foot; they never grew warm after the wound was received, notwithstanding every convenient mode of applying heat was resorted to for the purpose of restoring it. In about ten days his toes became black and apparently dry, and very soon the entire foot and leg, to within a few inches of the knee, became gangrenous and black. After using the whole catalogue of antiseptices, locally, I made deep incisions in the leg, but found neither sensibility nor blood.

On the 12th day of March, by the advice, and with the assistance, of my friend, Dr. Stahl, of Quincy, the limb was amputated above the knee, but below the ligated artery. His recovery from this operation was rapid and complete.

The question will doubtless be asked, why did gangrene of the foot and leg follow a laceration of the popliteal artery when there was no injury to the parts below the immediate vicinity of the knee? We cannot attempt to answer that question except upon the supposition that the popliteal nerve which, in that region, lies in contact, or nearly so, with the artery, was also lacerated. Thus by depriving the leg and foot simultaneously of blood and nervous influence, to the extent that evidently existed in this case, such a result might naturally be expected.

It was more than the simple injury done to the artery; the laceration must necessarily have interfered with the vessels which would under favorable circumstances, maintain the circulation by anastomosis. Gangrene would probably be the usual consequence in such cases.

AUGUST 17, 1853.

